# Molecular and Epidemiological Characterization of Emerging Immune-Escape Variants of SARS-CoV-2

**DOI:** 10.3389/fmed.2022.811004

**Published:** 2022-02-10

**Authors:** Kei Miyakawa, Sundararaj Stanleyraj Jeremiah, Yutaro Yamaoka, Takahiko Koyama, Reitaro Tokumasu, Michiharu Kudo, Hideaki Kato, Akihide Ryo

**Affiliations:** ^1^Department of Microbiology, Yokohama City University School of Medicine, Kanagawa, Japan; ^2^Life Science Laboratory, Technology and Development Division, Kanto Chemical Co., Inc., Kanagawa, Japan; ^3^International Business Machines Corporation Thomas J. Watson Research Center, New York, NY, United States; ^4^IBM Research - Tokyo, Tokyo, Japan; ^5^Infection Prevention and Control Department, Yokohama City University Hospital, Kanagawa, Japan

**Keywords:** SARS-CoV-2, emerging variants, neutralizing antibodies, dual antibody cocktail therapy, mRNA vaccine

## Abstract

The successive emergence of severe acute respiratory syndrome coronavirus 2 (SARS-CoV-2) variants has presented a major challenge in the management of the coronavirus disease (COVID-19) pandemic. There are growing concerns regarding the emerging variants escaping vaccines or therapeutic neutralizing antibodies. In this study, we conducted an epidemiological survey to identify SARS-CoV-2 variants that are sporadically proliferating in vaccine-advanced countries. Subsequently, we created HiBiT-tagged virus-like particles displaying spike proteins derived from the variants to analyze the neutralizing efficacy of the BNT162b2 mRNA vaccine and several therapeutic antibodies. We found that the Mu variant and a derivative of the Delta strain with E484K and N501Y mutations significantly evaded vaccine-elicited neutralizing antibodies. This trend was also observed in the Beta and Gamma variants, although they are currently not prevalent. Although 95.2% of the vaccinees exhibited prominent neutralizing activity against the prototype strain, only 73.8 and 78.6% of the vaccinees exhibited neutralizing activity against the Mu and the Delta derivative variants, respectively. A long-term analysis showed that 88.8% of the vaccinees initially exhibited strong neutralizing activity against the currently circulating Delta strain; the number decreased to 31.6% for the individuals at 6 months after vaccination. Notably, these variants were shown to be resistant to several therapeutic antibodies. Our findings demonstrate the differential neutralization efficacy of the COVID-19 vaccine and monoclonal antibodies against circulating variants, suggesting the need for pandemic alerts and booster vaccinations against the currently prevalent variants.

## Introduction

The rapid and nearly unrestricted global spread of coronavirus disease (COVID-19) has resulted in the evolution of various mutants of severe acute respiratory syndrome coronavirus 2 (SARS-CoV-2). With vaccines being the principal effective modality to curtail the pandemic, it is crucial to use them effectively and prepare for a rise in the number of immune-escape mutants that can evolve due to the selection pressure exerted. Based on their clinical and epidemiological significance, the World Health Organization (WHO) has identified variants of concern (Alpha, Beta, Gamma, and Delta), variants of interest (Lambda and Mu), and variants under monitoring (1). Although the Delta strain is the principal mutant responsible for the majority of the infections currently, variants with a few more amino acid substitutions in the Delta spike are emerging.

Previous studies have shown that mRNA vaccines such as BNT162b2 and mRNA-1273 confer robust protection against SARS-CoV-2 (2). However, several recent reports have shown that antibody titers drop markedly after 6–8 months of vaccine administration (3–6). However, there has been no temporal and comprehensive study of neutralizing activities against the increasing number of delta derivatives.

Several human monoclonal antibodies have been used for the treatment of COVID-19, which contribute to the reduction of viral load and symptoms (7, 8). However, some mutants have been shown to be resistant to these therapeutic antibodies, and the neutralizing capacity of the antibodies is greatly reduced (9, 10).

We recently developed a rapid neutralizing test, hiVNT, which enables the detection of SARS-CoV-2 neutralizing antibodies in sera within 3 h (11, 12). Therefore, by using hiVNT, we aimed to evaluate the efficacy of vaccine-derived neutralizing antibodies (nAbs) and therapeutic antibodies against the increasingly emerging recent variants.

## Materials and Methods

### Subjects and Ethics Statement

Participants were recruited from among the medical staff of Yokohama City University Hospital in March 2021. Written informed consent was obtained from all the participants. Blood samples were collected 1 week and 6 months after the administration of the second dose of Pfizer/BioNTech mRNA vaccine. Until the assessment date, we collected 126 one-week sera samples and 98 six-month sera samples, and all the samples were used. We randomly selected a set of 19 samples with blinding to demographic characteristics and designated this set as “Pvac19 sera panel.” Prior to the experiment, all samples were tested for antibodies against SARS-CoV-2 spike and nucleocapsid protein and were confirmed to be positive and negative, respectively (there was no previous/breakthrough infection). Blinding was not deemed necessary because the experiments did not involve any subjective assessment. No sample size calculation was performed. The study was conducted in accordance with the Declaration of Helsinki. This study was approved by the Yokohama City University Certified Institutional Review Board (Reference No. B210300001).

### Spike Haplotype Analysis

A total of 3,302,486 full genomes extracted from human subjects were downloaded from GISAID (13, 14) and the National Center for Biotechnology Information (NCBI) up to September 23, 2021. In total, 2,400,159 genomes met a data quality criterion of a < 200 bp gap. After a pairwise sequence alignment was performed with respect to the reference genome, we checked for improper alignments which induce artifactual frameshifts and removed such sequences from further analysis. Furthermore, we eliminated the hyper-variant samples with over 500 mutations. We did not observe any recurrent stop gain mutations in our analysis. Variant annotation was performed as described in our previous report (15). Briefly, a SARS-CoV-2 genome was first aligned in a pairwise manner against the NC_045512 reference genome using the Needleman-Wunsch algorithm (16) and differences from the reference genome were extracted as genome changes and subsequently annotated for the types of variants and for amino acid changes. A set of variants associated with amino acid changes in the spike protein were extracted for each genome. Such a set of variants was called the spike haplotype. Distinct spike haplotypes were identified from the entire set of genomes. Next, spike haplotypes were assigned to each genome, including the subset spike haplotypes. Therefore, a single genome could be classified into multiple spike haplotypes. For instance, a Delta variant spike haplotype consisting of T19R, 256_258delinsG, L452R, T478K, D614G, P681R, and D950N is also assigned to another haplotype group of T19R, L452R, T478K, D614G, P681R, and D950N, which is missing a 256_258delinsG variant. After grouping, the number of immune-escape variants, as reported previously (17–20), as well as the momentum, a metric of how quickly the frequency of a haplotype is increasing, were evaluated to identify the best candidates for antibody neutralization experiments.

### Rapid Neutralization Test (HiVNT)

hiVNT was performed as described previously (11, 12). Briefly, the target cells seeded in 96-well plates were inoculated with 50 μL of HiBiT-tagged virus-like particles (hiVLPs) containing diluted serum (1:20–1:43,740 dilution for the quantitative assay; 1:20 dilution for the qualitative assay). Intracellular luciferase activity was measured at 3 h after inoculation.

For the qualitative assay, the hiVNT score (percentage of luminescence signal inhibition) was calculated as follows:


(1) $$\begin{array}{l} \frac{\text{RLU }\left(\text{without serum}\right)-\text{RLU }\left(\text{with serum}\right)}{\text{RLU }\left(\text{without serum}\right)-\text{RLU }\left(\text{blank}\right)}\times 100\; \end{array}$$

For the quantitative assay, the dilution factor of serum that resulted in a 50% reduction in luminescence compared with that in the non-serum control was set as the hiVNT_50_. We calculated the hiVNT_50_ value using the curve-fitting tool ImageJ (NIH). When the serum exhibited no observable neutralizing activity to interpolate hiVNT_50_, it was assigned a hiVNT_50_ value of 10. Alternatively, cells were inoculated with 50 μL of hiVLPs containing diluted antibody (final concentration of 0.64–50,000 ng/mL for REGN-CoV2 and 0.32–25,000 ng/mL for LY-CoV). REGN-CoV2 and LY-CoV were research grade and were obtained from ProteoGenix and Invivogen, respectively. The concentration of the antibody that resulted in a 50% reduction in luminescence compared with that of the non-antibody control was set as EC_50_. The antibodies were tested individually, and the cocktail was considered effective against the viral mutant if it was neutralized by at least one antibody in the cocktail.

## Results

### Identification of Vaccine-Escape Variants

**Figure 1:**
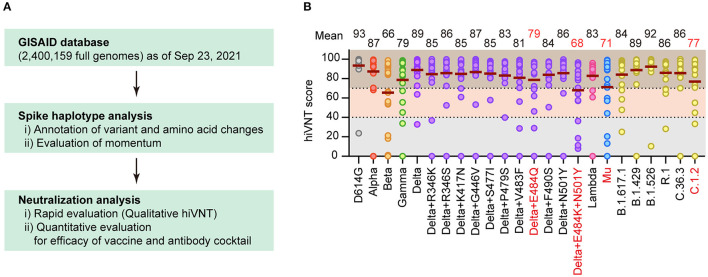
Identification of vaccine-escape variants. **(A)** Flow of this research. Spike haplotype analysis was used to search for mutants with specific mutations, and those with high growth rates were extracted and tested for neutralization. **(B)** Neutralizing activity of Pvac19 sera panel (*n* = 19, 1 week after the second dose) against each variant, calculated via a rapid neutralization test (qualitative hiVNT). The percentage of inhibition of viral infection by 20-fold dilution of serum is shown as the hiVNT score in the scatter plot. The mean of two independent determinations is plotted. The brown lines indicate the mean hiVNT scores, the values of which are displayed above the graph.

Of the 3,302,486 SARS-CoV-2 full genomes downloaded from GISAID on September 23, 2021, we selected 2,400,159 genomes that met the data quality criteria for the spike haplotype analysis. We identified 12,248 distinct spike haplotypes (i.e., sets of variants) with over 10 recurrences from the whole genome set using previously reported methods (15, 16). Based on the number of cases, the momentum, and immune escaping codons or mutations (17–20), we evaluated the number of immune-escape variants and the momentum to identify the best candidates for neutralization tests (Figure 1A).

To comprehensively identify the vaccine-escape strains, we performed a virus-like particle (VLP)-based rapid neutralization test (hiVNT) (11, 12) on post-vaccination sera collected from individuals one week after administration of the second dose of the BNT162b2 mRNA vaccine. In this study, a hiVNT score of 40 was set as the lower threshold, which is equivalent to 50% of the neutralizing titer against SARS-CoV-2 pseudovirus (pvNT_50_) >50, and a hiVNT score of 70 was set as the higher threshold (equivalent to pvNT_50_>200) (Supplementary Figure 1). These thresholds were decided based on a recent study reporting that the pvNT_50_ in sera of individuals with vaccine-breakthrough infections was approximately 200 (21). Samples that fell below the lower threshold were considered to exhibit no neutralizing activity, those between the lower and higher thresholds were considered to exhibit weak neutralizing activity, and those above the higher threshold were considered to exhibit strong neutralizing activity.

A “Pvac19 sera” panel (sera from 19 individuals collected one week after the second dose of Pfizer/BioNTech mRNA vaccine was administered) were used to determine the hiVNT score for each variant. The mean hiVNT score for most variants was approximately 80, indicating that the vaccine could induce sufficient levels of neutralizing antibodies against these mutants as well. However, four variants, namely Beta and Delta derivatives (Delta+E484Q, Delta+E484K+N501Y), Mu, and C.1.2, showed relatively low hiVNT scores (Figure 1B), suggesting that the neutralizing activity of post-vaccination sera against these variants might be weak.

### Neutralization of SARS-CoV-2 Variants by Vaccine Sera and Therapeutic Antibodies

**Figure 2:**
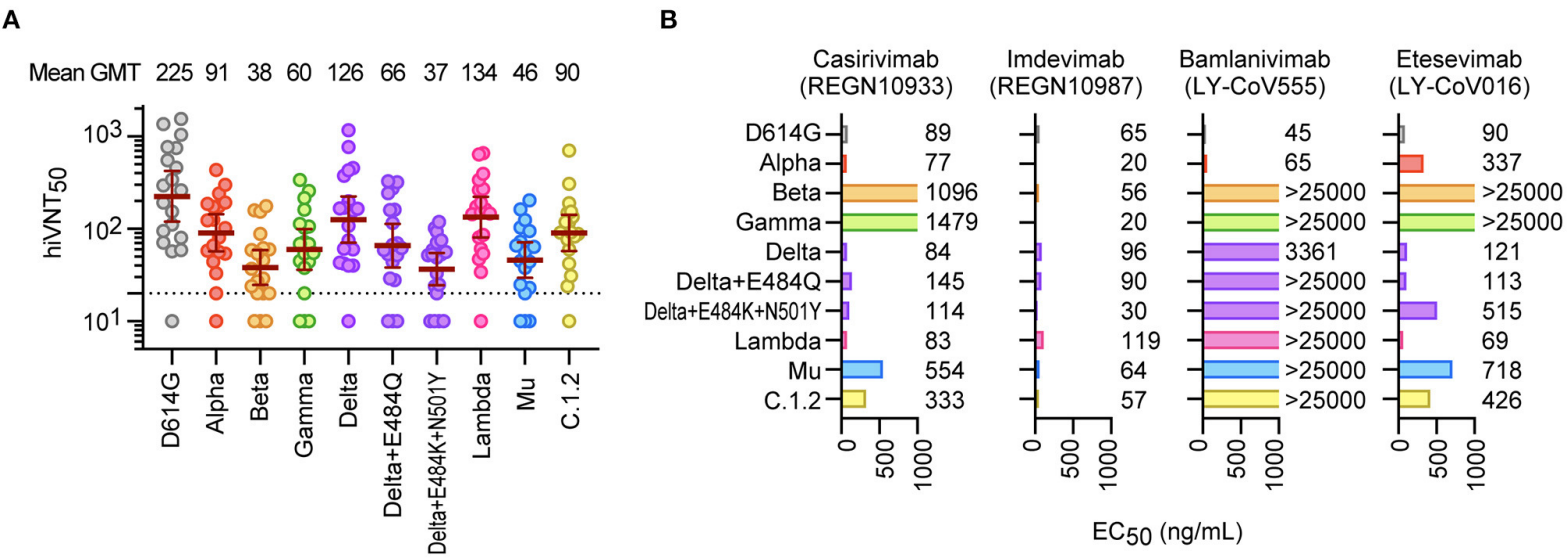
Neutralization of SARS-CoV-2 variants by mRNA vaccine sera and therapeutic antibodies. **(A)** Neutralizing activity of Pvac19 sera panel (*n* = 19, 1 week after the second dose) against each variant. Serum dilutions showing 50% inhibition of infection (hiVNT_50_) were determined via a quantitative hiVNT. The dotted line indicates the cut-off threshold of this assay (hiVNT_50_ = 20). The mean of two independent determinations is plotted. The brown lines indicate the geometric mean titers (GMT) ± 95% confidence intervals, the values of which are displayed above the graph. **(B)** Neutralization of each mutant strain by two dual antibody cocktails [REGN-CoV2; REGN10933 (Casirivimab) and REGN10987 (Imdevimab), and LY-COV; LY-CoV555 (Bamlanivimab) and LY-CoV016 (Etesevimab)]. The numbers indicate the 50% effective concentration (EC_50_, ng/mL), determined by two independent experiments. Since these nAbs are treated as a cocktail, they are considered effective if the EC_50_ of either antibody is equivalent to or lower than that of the D614G control.

Next, we quantitatively evaluated the neutralizing activity against these variants. The serum dilution factor that inhibits VLP entry by half (hiVNT_50_) was assessed to demonstrate the neutralizing activity of the sera against these variants. The geometric mean titers (GMTs) were 225 for D614G, 38 for Beta, and 37 for Delta + E484K + N501Y (Figure 2A), suggesting that the sera had 6-fold reduced neutralization efficacy against the Beta and Delta variants. However, the GMTs for all variants were above the effective threshold, suggesting that the vaccine-derived nAbs can neutralize the majority of variants tested.

We then evaluated the efficacy of the therapeutic antibodies (10, 22), REGN10933 (casirivimab), REGN10987 (imdevimab), LY-CoV555 (bamlanivimab), and LY-CoV016 (etesevimab), against these mutants. In the casirivimab/imdevimab combination, all tested mutants were found to be neutralized by at least one of the two antibodies in the cocktail (Figure 2B). In contrast, bamlanivimab and etesevimab were less effective, especially against the Beta and Gamma strains (Figure 2B). Etesevimab was still effective against Delta, but the effect was reduced in Delta + E484K + N501Y. We further demonstrated that the Mu variant can also cause cell–cell fusion, similar to the Delta variant (Supplementary Figure 2), which is highly likely to promote viral resistance to nAbs (23).

### Long-Term Analysis for Vaccine-Elicited Antibodies Against the Variants

We recently reported that neutralizing antibody titers drop to 20% at 6 months after vaccination (24). To examine the vaccine-elicited neutralizing antibody retention on a larger scale and over a longer period of time, we further increased the number of serum samples and compared the hiVNT scores of the variants at both 1 week (*n* = 126) and 6 months (*n* = 98) post-vaccination.

**Figure 3:**
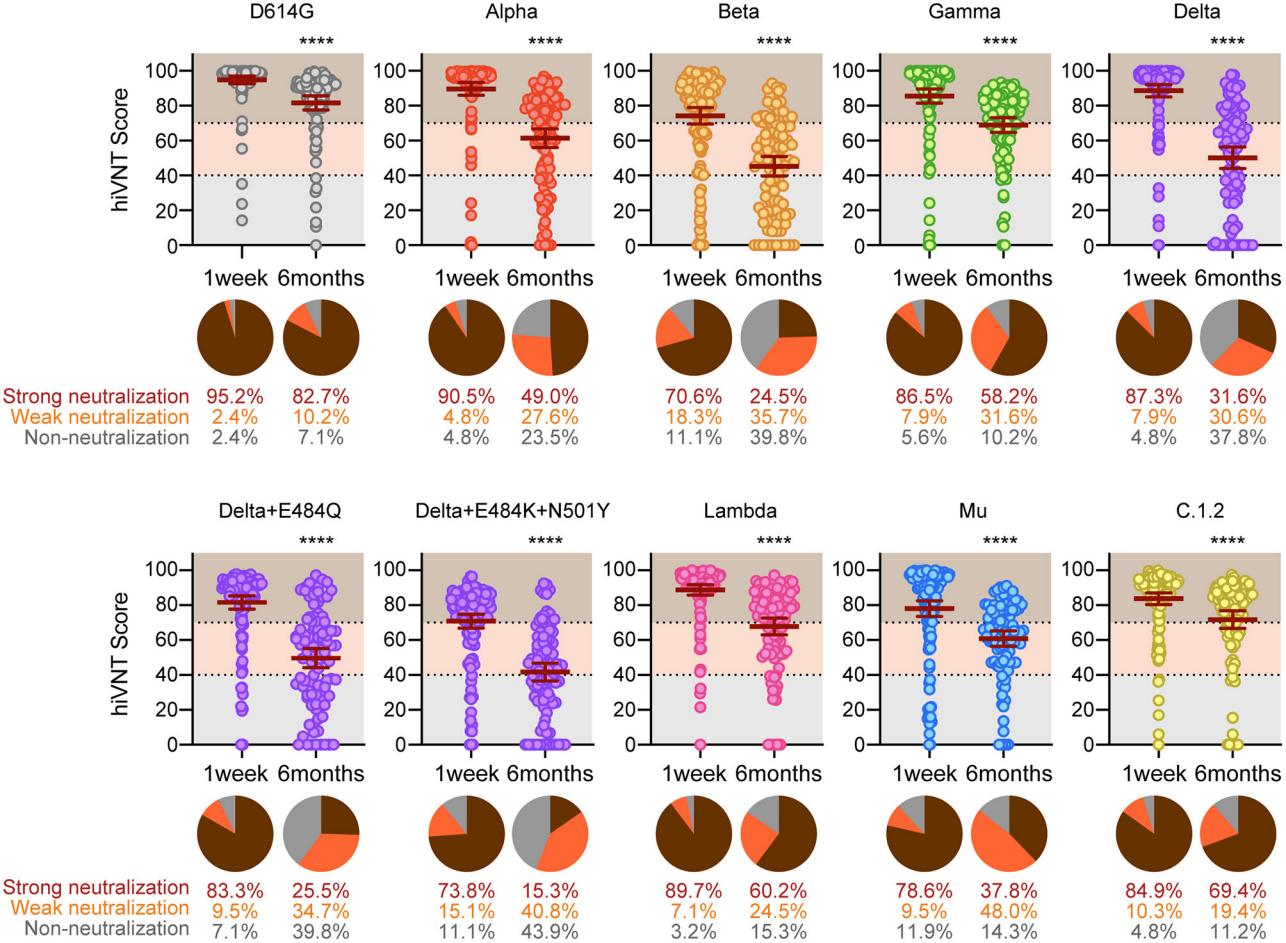
Long-term analysis for vaccine-elicited antibodies against the indicated strains. Positive rates for neutralizing antibodies determined via a qualitative hiVNT (*n* = 126 for 1 week and *n* = 98 for 6 months after the second dose) against the indicated variants. The mean of two independent determinations is plotted. The brown lines indicate the mean ± 95% confidence intervals. The percentage of neutralization potency based on the hiVNT score of each serum sample against the indicated variants is shown in the pie chart. An hiVNT score below 40 (equivalent to pvNT_50_ < 50) indicates non-neutralizing serum, a score of 40–70 (equivalent to pvNT_50_ > 50 but < 200) indicates weakly neutralizing serum, and a score above 70 (equivalent to pvNT_50_ > 200) indicates strongly neutralizing serum. See also Supplementary Figure 1 for a description of this definition. The mean of two independent determinations is plotted. ****P < 0.0001 (unpaired *t* test).

At 1 week after vaccination, strong neutralization (hiVNT score > 70) of all variants was observed in most of the sera samples, ranging from the highest (95.2%) in D614G to the lowest in the Beta variant (70.6 %) (Figure 3). Delta + E484K + N501Y and Mu showed a pattern similar to that of Beta, with 73.8% and 78.6% of the samples strongly neutralized, respectively. The proportion of sera samples that did not exhibit neutralizing activity was notably lower than that of those exhibiting neutralizing activity for each variant. The highest occurrence of nAb escape (including weak and non-neutralizing activity, i.e., hiVNT score < 70) was noted with Beta (29.4%), followed by Delta + E484K + N501Y (26.2%) and Mu (21.4%). This indicates that even immediately after two doses of mRNA vaccine, ~20–30% of vaccinees may be at a risk of breakthrough infection of these variants.

Our results indicated that, at 6 months after vaccination, 82.7% of the vaccinees exhibited strong neutralizing activity against the conventional strain. However, at 6 months after vaccination, strong neutralizing activity was significantly reduced against all mutant strains, ranging from the highest (60.2%) in the Lambda to the lowest in the Delta + E484K + N501Y variant (15.3 %) (Figure 3). This result suggests that the strong neutralizing activity against SARS-CoV-2 variants wane in 6 months after vaccination, yet a weak neutralization is present.

### Epidemiological Characterization of Vaccine-Escape Variants

**Figure 4:**
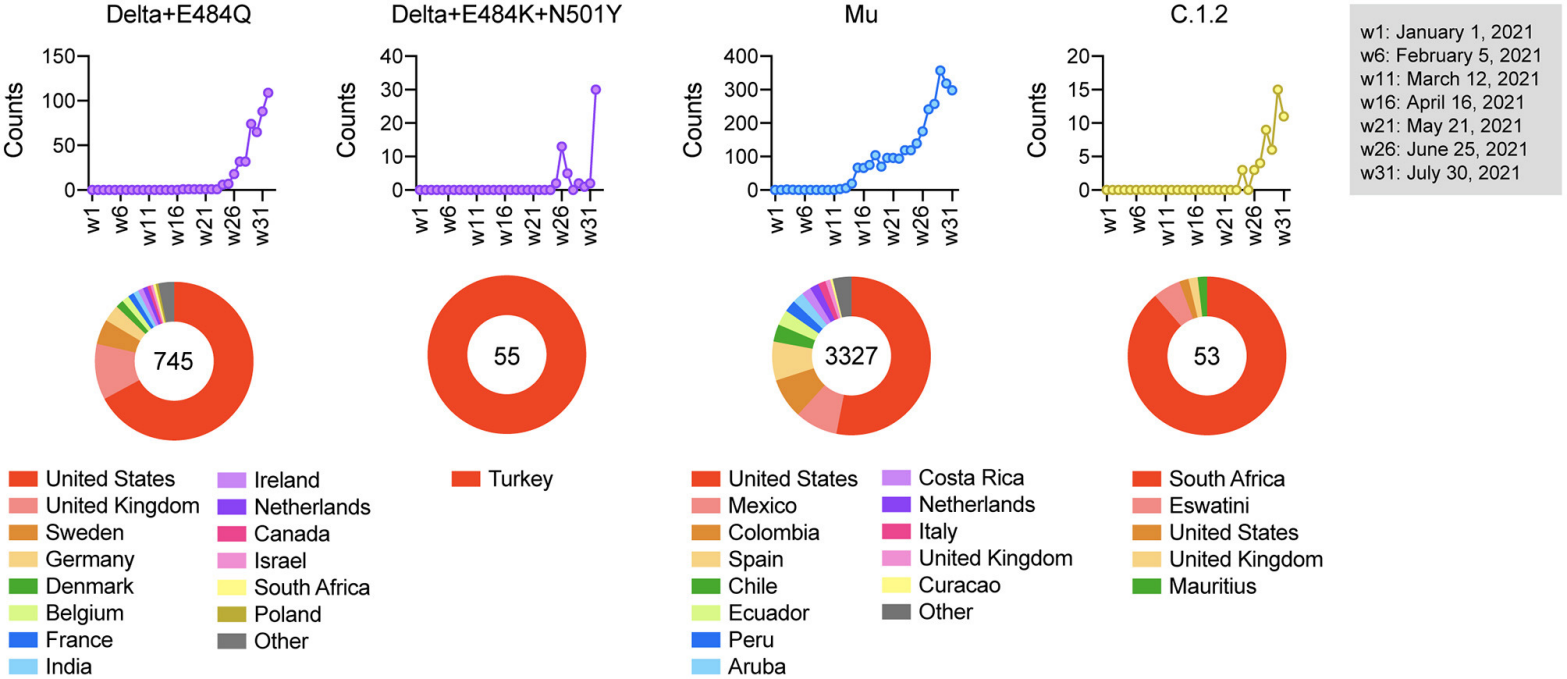
Epidemiological characterization of vaccine-escape variants. The upper graph shows the number of variants detected from week 1 to week 32 in 2021, and the lower pie chart shows the countries where the variants were detected. The numbers in the pie chart represent the number of detections.

Finally, we examined the regions in which these strains of concern were mainly detected. Our epidemiological analysis demonstrated that the frequency of Delta + E484Q increased since week 24 of 2021 and the strain is still detected worldwide. Delta + E484K + N501Y was detected only in Turkey from week 26, Mu was prevalent in South America from week 14, and C.1.2 was prevalent in South Africa from week 26 (Figure 4). The vaccination status in these countries is shown in Supplementary Figure 4. We noticed that many of these haplotypes emerged before widespread vaccination, suggesting that vaccination might not be the likely cause of this emergence. Since vaccine-induced humoral immunity is less effective against these variants, their spread needs to be monitored carefully.

## Discussion

In this study, by combining haplotype analysis and hiVNT, we identified immune evasion variants that showed an increasing local trend. In addition, we tested the long-term efficacy of the Pfizer/BioNTech mRNA vaccine against these variants.

With the rise in emerging variants such as Delta derivatives, Mu, and C.1.2, concerns regarding the efficacy of the currently available vaccines and antibody cocktail therapy have emerged. Our results show that the vaccine-derived nAbs and the antibody cocktail exhibit neutralization efficacy against these variants. We observed this effect in the sera of vaccine recipients shortly after the administration of the second dose when the nAbs were considered to be at peak levels.

As vaccine-derived nAbs wane over time, follow-up studies are necessary to assess the persistence of nAbs against these variants. In fact, our analysis using sera 6 months after vaccination showed that the positive rate of nAb against the conventional strain was relatively maintained, while that against the mutant strains was markedly decreased. In particular, only about 15–30% of vaccinees showed potent neutralizing activity against Delta, Delta + E484K + N501Y, and Mu strains. A comprehensive depiction of antibody prevalence by a hiVNT mutant panel not only allows for a rapid assessment of vaccine-elicited humoral immunity, but also highlights the need for booster vaccinations in areas where the mutant strains are prevalent.

Several reports have shown that after 2–3 months of vaccination, the neutralizing activity on variants such as Delta strains is significantly lower than that of WT (25). We have also shown a faster time-bound deterioration in neutralizing activity against the Delta strain (38% negative for neutralizing activity) than the WT strain (7% negative for neutralizing activity) in 6 months after vaccination, and this may be a major factor in breakthrough infections caused by Delta.

Analysis of the therapeutic antibodies against the variants showed that imdevimab had high neutralizing activity against all the mutants tested, but casirivimab had reduced activity against Beta, Gamma, and Mu. These strains commonly include the E484K mutation, and this mutation is considered to be a limitation associated with casirivimab, as previously indicated (10). Unfortunately, bamlanivimab showed no neutralizing activity against the variants except Alpha, suggesting that it is ineffective against the current prevalent strains. Etesevimab showed absolutely no neutralizing activity against Beta and Gamma, consistent with a previous report (9), and we found that this mAb was less effective against other mutant strains besides Delta and Lambda. The N501Y mutation was common in the strains with reduced efficacy, suggesting that this mutation is a limitation of etesevimab.

Our results show that the Delta derivatives possess a higher vaccine escape than their parent Delta strain. Likewise, the Mu variant possesses a higher vaccine-escape ability than the Delta variant and also exhibits cell–cell fusion property like the latter. In general, an increased cell-cell fusion capacity indicates a high concentration of virus (or spike) in the fusion zone, and a relatively high concentration of nAbs is required to prevent the infection of neighboring cells (26). Therefore, such viral strains are more likely to evade humoral immunity. Hence, these variants could present a major challenge if either or both, or other immune escape mutants progresses to replace the Delta variant as the most predominantly transmitted variant. In the future, vaccines and therapeutic antibodies should be designed to address this problem.

## Data Availability Statement

The data analyzed in this study is subject to the following licenses/restrictions: The list of analyzed genomes from the GISAID's EpiFlu database is available from the corresponding author upon reasonable request. Requests to access these datasets should be directed to Kei Miyakawa, keim@yokohama-cu.ac.jp.

## Ethics Statement

This study was approved by the Yokohama City University Certified Institutional Review Board (Reference No. B210300001). The patients/participants provided their written informed consent to participate in this study.

## Author Contributions

KM designed and performed the research, interpreted the data, and wrote the manuscript. SJ and RT interpreted the data and wrote the manuscript. YY performed the research and interpreted the data. TK designed and performed the spike haplotype analysis, interpreted the data, and wrote the manuscript. MK interpreted the data. HK collected the specimens. AR directed the research, interpreted the data, and wrote the manuscript. All authors contributed to the article and approved the submitted version.

## Funding

This study was supported by AMED grants (JP20he0522001 and JP21fk0108104) to AR.

## Conflict of Interest

YY is a current employee of Kanto Chemical Co., Inc. TK, RT, and MK are current employees of IBM. The remaining authors declare that the research was conducted in the absence of any commercial or financial relationships that could be construed as a potential conflict of interest.

## Publisher's Note

All claims expressed in this article are solely those of the authors and do not necessarily represent those of their affiliated organizations, or those of the publisher, the editors and the reviewers. Any product that may be evaluated in this article, or claim that may be made by its manufacturer, is not guaranteed or endorsed by the publisher.
