# Supplementary material for: Molecular and Epidemiological Characterization of Emerging Immune-Escape Variants of SARS-CoV-2
Source: Front Med (Lausanne). 2022 Feb 10;9:811004. doi: 10.3389/fmed.2022.811004 (PMC8866700; doi:10.3389/fmed.2022.811004)
Supplement: Supplementary file 1 [file Data_Sheet_1.pdf]

Supplementary Material

Supplementary Figures

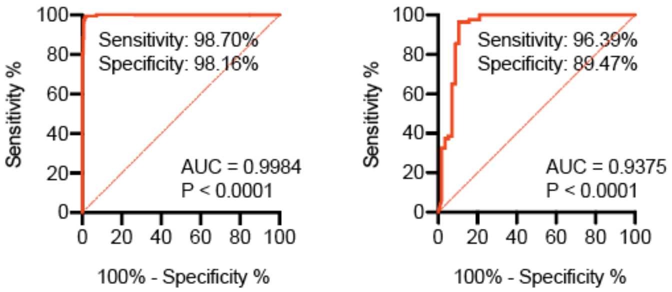

Supplementary Figure 1. Accuracy of qualitative hiVNT scoring and neutralization activity

The left panel shows the receiver operating characteristic (ROC) curve when a hiVNT score of 40 was defined as a nAb-negative ($pvNT_{50}$<50) in the pseudovirus neutralization test. The right panel shows the ROC curve when a hiVNT score of ≥70 was defined by strong neutralizing activity ($pvNT_{50}$>200). Although the qualitative hiVNT method measures only a 20-fold dilution of serum, it provides semi-quantitative results [note that the area under the curve (AUC) was greater than 0.90 in both cases].

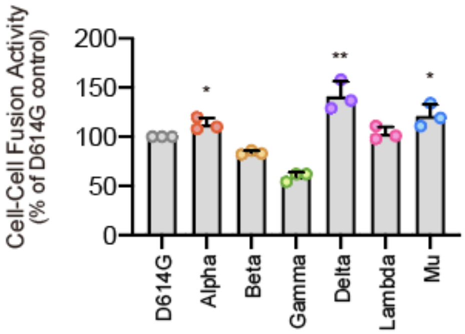

Supplementary Figure 2. Cell–cell fusion activity of the SARS-CoV-2 variants

The assay was performed using a split luciferase system. HEK293-donor cells (expressing SARS-CoV-2 spike and HiBiT) and HEK293-acceptor cells (expressing ACE2 and LgBiT) were co-cultured in a 1:1 ratio. After 3 h, luciferase activity associated with cell–cell fusion was measured. *P < 0.05, **P < 0.01. A higher luciferase signal indicates a higher occurrence of cell–cell fusion.

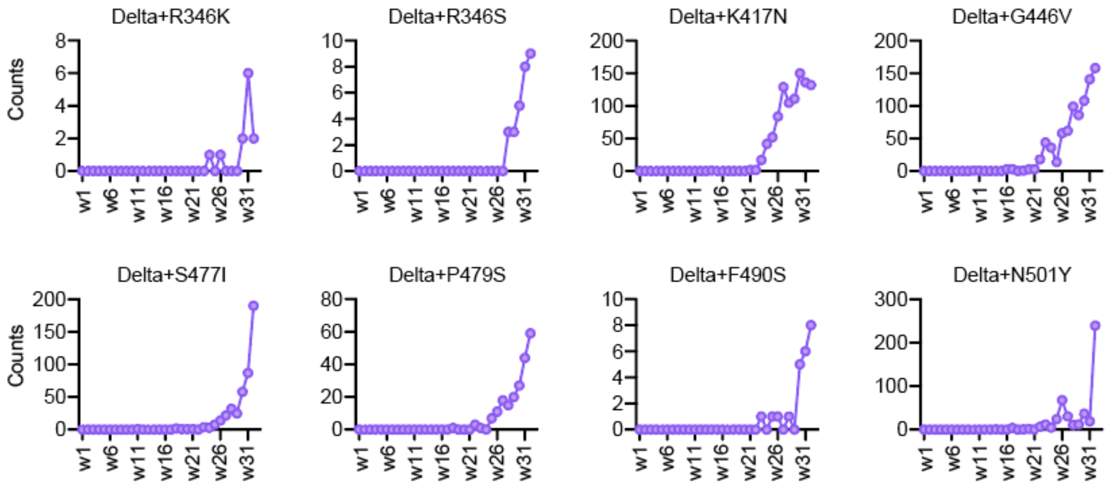

Supplementary Figure 3. Epidemiological characterization of the Delta derivatives

The graph shows the number of variants detected from week 1 to week 32 in 2021.

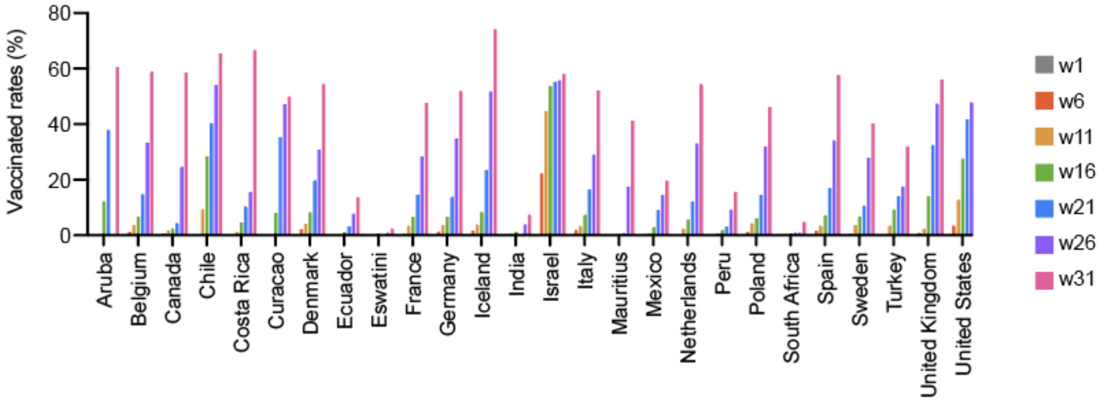

Supplementary Figure 4. Vaccinated rates of indicated countries

The graph shows the vaccinated rates of indicated countries from week 1 to week 31 in 2021, collected from the public database (https://ourworldindata.org/covid-vaccinations).
